# Experimental Investigation on the Effect of Converter Slag Aggregate for Blended Mortar Based on CT Scanning

**DOI:** 10.3390/ma14247570

**Published:** 2021-12-09

**Authors:** Min Jae Kim, Woong Ik Hwang, Won Jung Cho

**Affiliations:** 1 Department of Civil, Environmental and Architecture Engineering, Korea University, Seoul 02841, Korea; manta209@korea.ac.kr; 2 Department of Civil and Environmental System Engineering, Hanyang University, Ansan 15588, Korea; nakta83@hanyang.ac.kr

**Keywords:** ferronickel slag, pulverized fly ash, converter slag, industrial by-products, pozzolanic reaction, resource recycle, soundness effect, X-ray CT technique

## Abstract

This study investigated the air aging converter (Basic Oxygen Furnace, BOF) slag aggregate mortar with pulverized fly ash (PFA) and ferronickel slag (FNS). The chemical composition and mineralogical constituents of BOF incorporated mortar were analyzed. Setting time, flowability, compressive strength, and length change were measured to evaluate the fundamental properties of BOF mortar. The X-ray CT analysis was employed to observe the effect of converter slag in the cement matrix visually. The results showed that the hydration of BOF generated a pore at the vicinity of the aggregate, which decreased the compressive strength and increased the length change of mortar. However, the PFA or FNS incorporation of PFA or FNS can decrease the alkalinity of pore solution and subsequently reduce the reactivity of BOF aggregate. Thus, the incorporation of PFA and FNS can be a way to eliminate the disadvantage of BOF, such as volume expansion.

## 1. Introduction

The cement industry grows at 6% every year [1] because concrete is used as construction material worldwide. However, although cement is a necessary construction material, the cement industry is considered one of the most energy-intensive industries [2,3]. Additionally, about 5% of the carbon dioxide is produced by the cement industry. According to several reports, roughly 900 kg CO_2_ is produced per ton of cement [1,2,3,4]. In line with this situation, global environment load reduction policies, such as carbon emission rights, were enforced a clean development mechanism were enforced. Nevertheless, it is estimated that 5.8 billion tons of cement will be produced by 2050 [5]. Therefore, the development of supplementary cementitious materials (SCMs), derived from the by-product industry, have been arising as an alternative way to reduce the required amount of cement production.

Compared to the exhaustive research of SCMs, however, the deficiency of natural aggregate is still an exigent issue. Aggregate is an indispensable component in concrete, occupying nearly 65–80% of the total volume, whose effects on concrete performance are rarely studied [6]. Due to their inert and impervious characteristics, the durability of concrete is affected by the quality of aggregates. According to [7], the worldwide market for construction aggregate was projected to advance to 51.7 billion in 2019, representing an annual growth rate of 5.2%. Furthermore, sand and gravel reserves are shrinking across much of the world, and illegal sand mining plagues a number of developing markets that have rapidly growing sand consumption requirements [7]. To meet this global demand for concrete in the future, it is becoming a more challenging task to find suitable alternatives to natural aggregate for preparing concrete [8]. Hence, the use of alternative sources for natural aggregates is becoming increasingly important.

As given in Table 1, steel slag is a vast majority of solid wastes, produced as a by-product during the steel-making process. It is reported that 18,666,000 tons of steel slag were generated in 2007, an increase of 6.5% compared with the previous year [9,10,11]. Currently, the annual emission of steel slag in China is 101 million tons [12], with 43.43 million tons in Korea [13]. This steel slag can be classified according to the smelting process, as shown in Table 1. Among them, standards for using slag as a construction material have also been established [14,15], except for converter slag.

Converter slag is a by-product of steelmaking produced during the conversion of pig iron into steel in a basic oxygen furnace. This solid waste has variable oxide composition with a high amount of CaO, Fe_2_O_3_ with minor oxides of SiO_2,_ and MgO [16], which are also present in ordinary Portland Cement (OPC). Despite these similar mineral phases of converter slag compared to OPC, its utilization is very low due to the presence of free lime (CaO) and free MgO, which can induce unstable volume expansion. For these reasons, most discharged converter slag is dumped away or backfilled. To overcome this limitation, a few studies using steel slag including converter slag as a construction material were conducted [17,18]. According to [17], sufficiently stabilized steel slag aggregate by hot water or steam aging might have potentialities in concrete aggregate by decreasing the expansion. Moreover, the steel slag aggregate shows to be beneficial for aggregate substitution for road paving applications [18]. It was also reported that the partial replacement of cement by ground steel slag powder improved the ductility of cement-based materials [19]. However, there are still many concerns about cementitious reactions of converter slag under ambient conditions of hydration. Especially, the analysis of the behavior of converter slag aggregate in the cement matrix was not clearly covered. Most of the literature [17,20] observed the expansion of the specimens based on experiments on fundamental properties such as length change rate and setting time, not visually. Besides, the literature is limited only to the cement matrix, a method of treatment at high temperature. There is no clear information about soundness mechanism of converter slag in blended cement.

This study investigated the air aging converter slag aggregate mortar with pulverized fly ash (PFA) or/and ferronickel slag (FNS), which is currently emerging for its feasibility as a cement admixture. The chemical composition was measured by X-ray fluorescence (XRF), while the mineralogical constituent was analyzed by X-ray diffraction (XRD). To observe hydrated phase composition, SEM/EDX analysis was also conducted. Flowability, compressive strength, and length change were also measured to detect fundamental properties of mortar. Subsequently, X-ray CT analysis was employed to visually evaluate the effect of incorporation of converter slag into the cement matrix.

## 2. Materials and Methods

### 2.1. Materials

**Figure 1:**
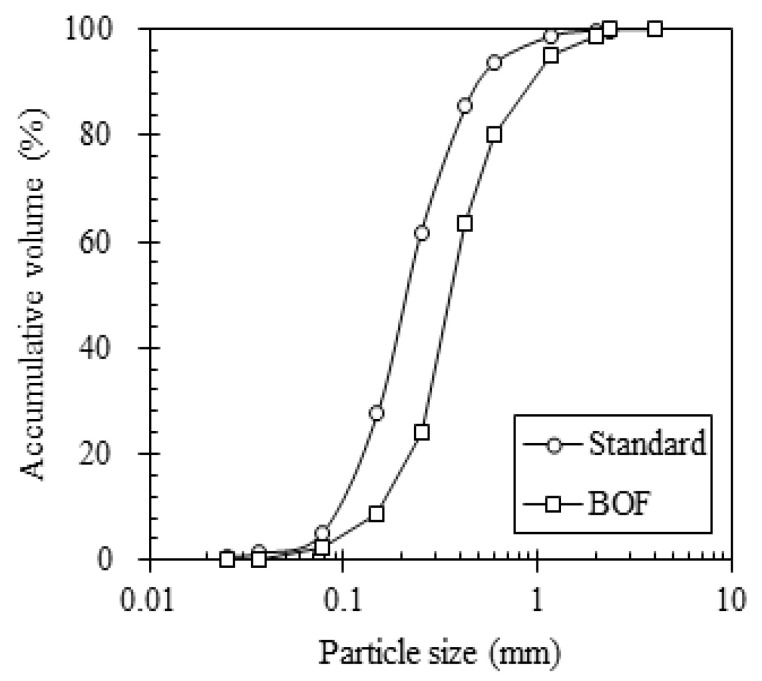
Particle size distribution of sand and BOF slag.

The converter slag, also known as Basic Oxygen Furnance (BOF) slag, used in this experiment was derived from a steel company in Chungnam, Korea. In this plant, the BOF slag was sieved to 4.75 mm and treated by the air aging method for 3 months under atmospheric conditions in order to reduce the expansibility. The particle size distribution (PSD) of natural sand and BOF slag was determined using the laser diffraction method (CILAS 1064), plotted in Figure 1.

The chemical compositions of OPC, PFA, FNS, and BOF slag, which were analyzed by XRF, are shown in Table 2. The main chemical components of the FNS powders were SiO_2_, MgO, and Fe_2_O_3_. The CaO contents of FNS and PFA were counted for only 6.28% and 3.93%, which was lower than OPC and BOF slag. According to the chemical composition of PFA, shown in Table 2, it can be classified as class F [21], which is principally composed of silicon with minor amounts of aluminum oxide. The main oxide compositions of the BOF slag were CaO and SiO_2_. However, BOF slag showed the highest content of Fe_2_O_3_ compared to other binders. Clearly, BOF slag clinker composition was more similar to OPC than highly siliceous materials (i.e., PFA and FNS).

**Figure 2:**
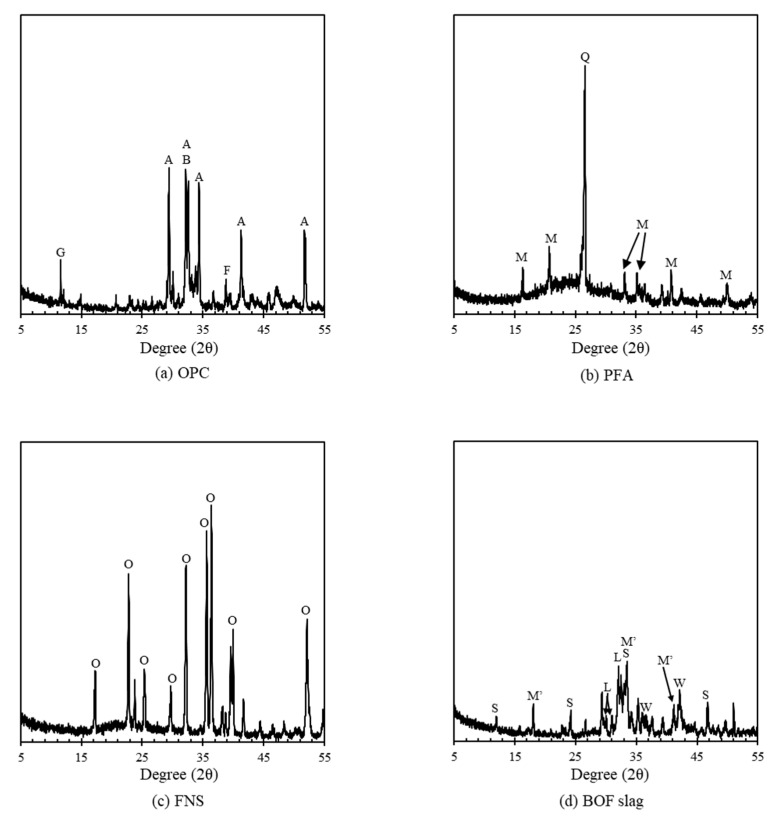
XRD curves of raw materials modified from [22] (Reproduced with permission from Materials; published by MDPI, 2020); (**a**) OPC, (**b**) PFA, (**c**) FNS, (**d**) BOF slag. A: Alite (C_3_S), B: Belite (C_2_S), F: Ferrite (C_4_AF), G: Gypsum (CaSO_4_), Q: Quartz (SiO_2_), M: Mullite (Al_2_Si_6_O_13_), O: Olivine (Mg, Fe)_2_SiO_4_, W: Wustite (FeO), S: Srebrodolskite (Ca_2_(Fe^+3^)_2_O_5_, M’: Mayenite (Ca_12_Al_14_O_33_), and L: Larnite (Ca_2_SiO_4_).

Figure 2 presents the XRD curves of raw materials. A detailed test method is suggested in Section 2.3. In the case of OPC, alite (C_3_S, 3CaO∙SiO_2_) and belite (C_2_S, 2CaO∙SiO_2_) were the main clinkers, while other clinkers such as gypsum, periclase, and brownmillerite (4CaO∙Al_2_O_3_∙Fe_2_O_3_) accounted for lower quantities. The XRD patterns showed high silica contents for PFA, while the main composition of FNS accounted for forsterite (Mg_2_SiO_4_) and fayalite (Fe_2_SiO_4_), which have a crystalline nature [22]. The forsterite showed a late hydration property, which takes about 2 years for complete reaction [23]. The BOF slag was mainly composed of crystalline clinker such as wustite (FeO), srebrodolskite (Ca_2_(Fe^+3^)_2_O_5_, mayenite (Ca_12_Al_14_O_33_), and larnite (Ca_2_SiO_4_). Although the main clinker of BOF slag included similar silicates’ clinker to that of OPC, larnite has a slower hydration rate rather than the major compound of OPC such as C_2_S (Ca_3_SiO_5_). Thus, the cementitious reactions of BOF slag are not evident under normal conditions of hydration [20].

**Figure 3:**
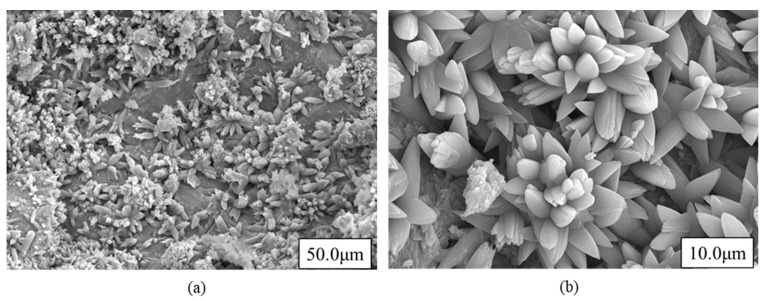
SEM image of BOF slag aggregate with different magnification; (**a**) 50.0 μm, (**b**) 10.0 μm magnification.

From the SEM images in Figure 3, the crystalline particles constitute BOF slag. It can be assumed that anhydrous substrate of free lime (CaO) was modified into the product of calcite during air aging exposure.

### 2.2. Mix Proportion

In fabricating BOF slag aggregate specimens, the replacement ratio of the admixture was taken as 30% of the total binder. A detailed mix proportion for each specimen is tabulated in Table 3. The water-to-cement ratio was kept at 0.45 irrespective of mix type. To figure out the chemical reactivity and fundamental properties, mortar specimens were employed. The specimens were demolded within 24 h after casting and then cured in a damp chamber at 23 ± 2 °C, RH 95%. The experiments were conducted at an ambient condition at 23 ± 2 °C, RH 50 ± 5%.

### 2.3. Fundamental Properties

#### 2.3.1. Flowability

To identify workability of the unhardened BOF slag mortar, a flow test was performed, according to ASTM C1437-15, after the mixing process. The slump cone was placed on the center of a table instrument, and then the mortar was poured slowly with tamping until the cone was sufficiently filled. After filling, the slump cone was lifted and the flow table was dropped 25 times in 15 s to allow the spread of the mortar. At the time of 15 s since lifting the cone, the diameters of the spread mortar were measured in two orthogonal directions and the average value was taken as the spread of the mortar.

#### 2.3.2. Setting Time

The setting time was measured by the penetration resistance of unhardened cement paste, according to ASTM C 191, using VICAT apparatus followed by a standard test specimen. After mixing, the fresh mortar was poured into a VICAT mold immediately and kept in an ambient condition (RH23 ± 2%). After filling, the mold was slightly shaken to remove the air and the surface of the mortar was smoothened. The initial and final sets were defined as the time to reach 3.5 MPa and 28.0 MPa of the penetration resistance.

#### 2.3.3. Compressive Strength

The compressive strength was measured by using the mortar specimens (50 mm × 50 mm × 50 mm) at 3, 7, 28, and 90 days, following ASTM C39. The replication of each measurement was five, and the average value was taken as the compressive strength at the corresponding age.

#### 2.3.4. Length Change

To measure autogenous shrinkage, prism mortar specimens (40 mm × 40 mm × 160 mm) were prepared in accordance with KS F 2586. Fresh mortar was cast into prism molds and consolidated to reduce voids and entrapped air in the mix. To prevent moisture loss, mortars were covered by a polytetrafluoroethylene sheet for the first 24 h. Then, the prism mortars were kept in a dry condition at atmospheric temperature (100% RH; 23 ± 0.5 °C), driving an evaporation of water, which may cause an internal strain and consequently change the volume of specimen. A strain gauge for measuring autogenous shrinkage was installed at the center of the specimen in the longitudinal direction and monitoring was conducted for 35 days.

### 2.4. Hydration Reactivity

#### 2.4.1. X-ray Diffraction (XRD)

All mineral admixtures and rushed and sieved (100 μm) mortar powders containing BOF slag aggregate were tested using XRD. The powder samples derived from the 90-day matured BOF slag mortars were investigated with the XRD technique. The scanning was applied in the diffraction range (2θ) of 5–60°at a scan rate 2.0°/min with 40-kV voltage and 100-mA current. The software package MDI JADE was used to mineralogically determine the hydration products. All powder samples were analyzed using XRD immediately after grinding of the specimen to avoid carbonation.

#### 2.4.2. SEM Image

After 28 days of curing, the surface of the mortar specimen was polished with sandpaper (up to 2000 grit) for the Scanning electron microscope (SEM). SEM with energy dispersive X-ray spectra (EDS) was acquired using a S-4800 (Hitachi) at 15-kV accelerating voltage and working distance = 11–13 mm; magnification of ×10,000 was performed on crushed mortar specimen after 90 days of curing. Thirty analyses were carried out for each sample, and each spectrum was collected until exposure time reached to 1000 counts in the intensity.

### 2.5. X-ray Computed Tomography

**Figure 4:**
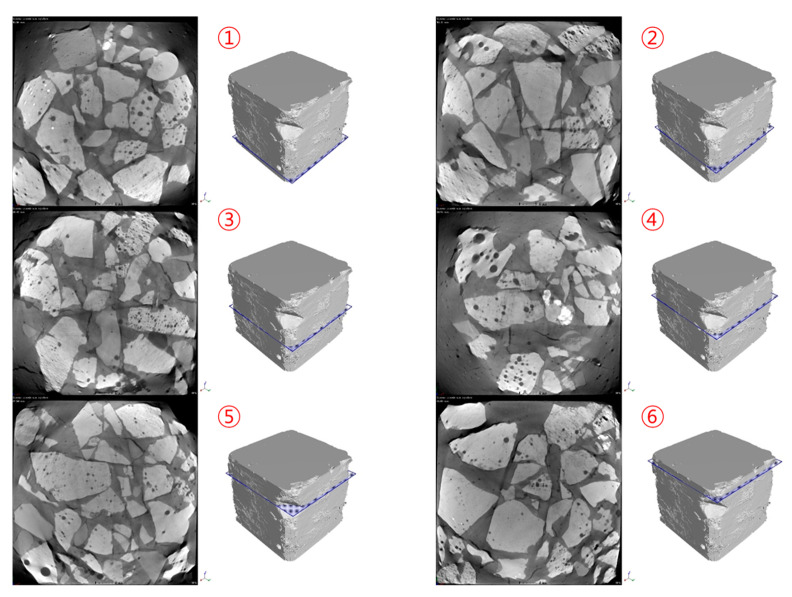
CT scanning process of mortar.

X-ray computed tomography analysis was conducted to visually observe the expansion behavior of the converter slag aggregate. After 90 days of curing, the mortar specimens (50 × 50 × 50 mm) were kept at ambient temperature for 1-h stabilization of mass. Then, the sample was placed in the CT scanning equipment. The test setup was derived and modified based on the literature [24]. The details of equipment and operation are given in Table 4 and Figure 4, and the directional target was applied in this study.

(1) X-ray Detector specification was as below:

Type: Digital flat panel detector, Radiation energy: 40 ~ 320 kV, Active area: 400 mm (h) × 400 mm (v)

Pixel matrix: 1024 (h) × 1024 (v), Pixel pitch: 200 µm, Resolution: 2.5 lp·mm@15 FPS(1 × 1), 1.25 lp·mm@30 FPS(2 × 2).

(2) X-ray Detector specification was as below:

Max. size of 3-D CT scanning: Ø 300 mm × 900 mm (h), Repetition accuracy: 0.004°.

After scanning, the obtained images were filtered to remove the noises and segment the pores using the OTSU (otsu algorithm) [25]. Then, we reconstructed the obtained images into a 3D image in Avizo software. Detailed scan conditions are described in Table 5.

## 3. Results and Discussion

### 3.1. Fluidity and Setting of BOF Slag Mortar

The variation of blended mortars’ flow and setting time is depicted in Table 6. The diameter for OFA0 accounted for 170 mm while the values were increased to 176 and 181 mm for OFA15 and OFA30, respectively. Although there was no significant difference between specimens, it was confirmed that incorporation of SCM increased the fluidity of BOF slag mortar. Unlike the test result of flowability, the result of setting time showed a considerable difference. It showed that the penetration resistance was achieved much faster for a lower substitution of SCMs in mortar. The initial set for OFA0, OFA15, and OFA30 was 195, 260, and 314 min, while 245, 382, and 454 min were for the final set, respectively. The delayed hardening process of fresh mortar may have arisen from the spherical shape of PFA, which has a ball-bearing effect that allows cement to be produced using less water; the higher replacement of PFA increased the flowability of the mortar.

### 3.2. Hardened Mortar

**Figure 5:**
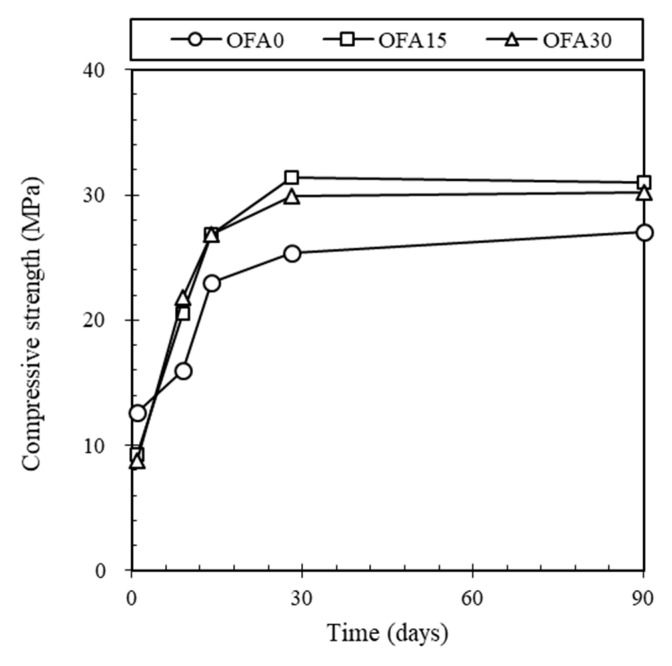
Development of compressive strength of BOF mortar.

Figure 5 presents a development of the compressive strength for BOF slag mortars at different curing ages. The compressive strength of OFA0 showed the lowest value at all curing ages except 1 day. The addition of PFA or/and FNS increased the compressive strength of the BOF slag mortar. For example, the early age strength of OFA0 at 9 days was 16.02 MPa, while OFA15 and OFA30 indicated 20.49 and 21.85 MPa, respectively. As curing age increased, OFA15 and OFA30 showed a gradual increase in strength, up to 30.95 and 30.17 MPa at 90 days while OFA0 reached 27.00 MPa.

From the compressive strength test results, it was notable that the compressive strength of BOF slag mortar was lower than that of mortar using natural sand. It is well known that the 28 days’ strength of mortar using natural sand is generally 30–40 MPa. However, only OFA15 exceeded 30 MPa, and OFA0 showed about 24.7 MPa. Based on the research from Yung-Chin Ding et al. [26], It was found that BOF showed lower strength than natural sand when it was used as an aggregate. Additional strength development was expected due to the slow hydration of larnite (β-Ca2SiO4) in BOF [20]. However, this occurs with more than 150 days of curing, which cannot be expected from the results of this study. According to the compressive strength test results of OFA15 and OFA30, the use of PFA or FNS can compensate for the strength reduction caused by BOF. This gain in strength may be attributed to a latent or pozzolanic reaction of FNS and/or PFA.

**Figure 6:**
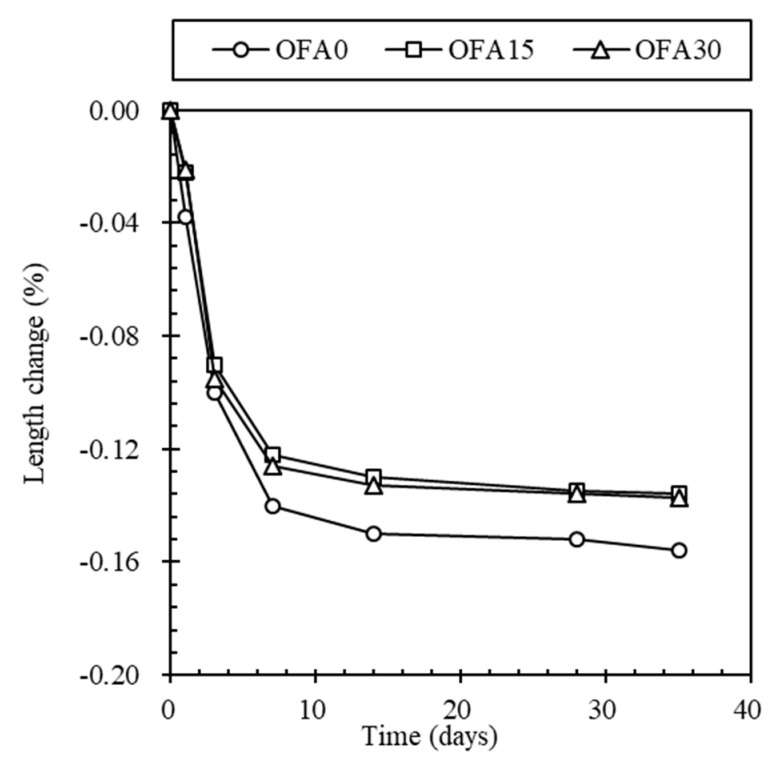
Length change of BOF slag mortar for 35 days after casting.

The length change measurement of BOF slag mortar was conducted to indicate hydration reactivity, as shown in Figure 6. Length change of OFA0 was always higher than that of OFA15 and OFA30, and the rate of all types significantly increased for 7 days then converged slowly. The value of length changes for the accelerated at 7 days for OFA0, OFA15, and OFA30 were accounted for as −0.140%, −0.122%, and −0.126%, while it was −0.156%, −0.136%, and −0.137% at 35 days, respectively. Notably, the change rate for OFA15 was mostly identical to OFA30, while the OFA0 indicated a significant increase in the length change by −0.014% at 7 days and −0.019% at 35 days. The increased volume change of OFA0 mortar may be derived from the reactivity of free lime in BOF slag, limiting its application in the construction industry. With higher use of OPC, the more undesirable hydraulic reaction would occur in the BOF slag mix, resulting in soundness volume change. However, replacement of PFA or/and FNS showed different properties of length change: Delayed setting time, increased flowability, higher mortar strength, and a lower rate of length change were gained for OFA15 and OFA30 compared to OFA0. The different properties of fresh mortar of OFA15 and OFA30 seemed to be attributed to the substitution of SCMs in the mix, which could have less CaO as cement clinker and less formation of Ca(OH)_2_ in cement mix so that free lime in BOF slag enabled it to produce hydrates, especially Ca(OH)_2_. Notably, the ternary-blended mortar of OFA15 showed more enhanced fundamental properties than OFA0 and OFA30; thus, it is more feasible for BOF mortar mix.

### 3.3. Hydration Products

#### 3.3.1. XRD Analysis

**Figure 7:**
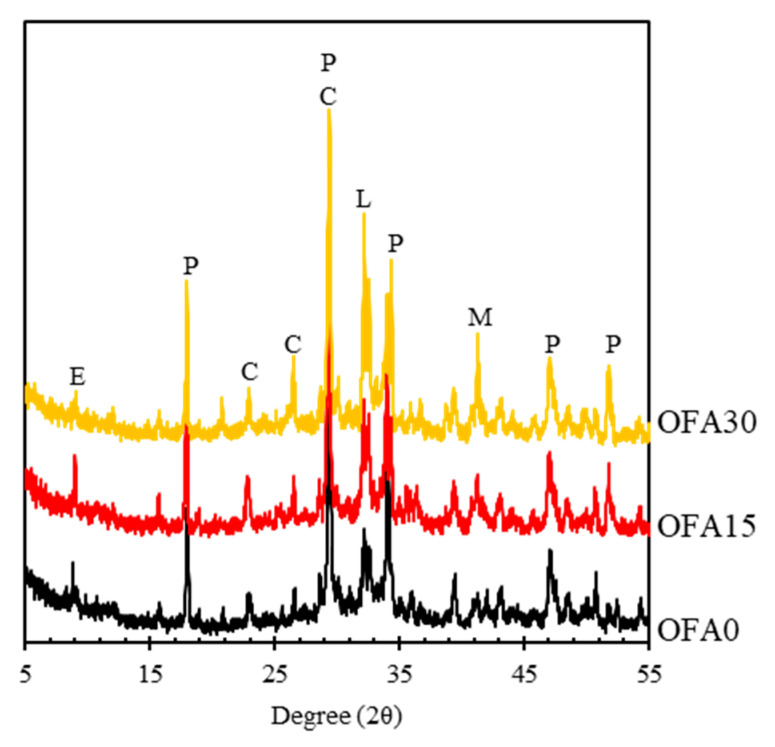
XRD curve for BOF slag mortar at 90 days of curing. (E: Ettringite Ca_6_Al_2_(OH)_12_(SO_4_)_3_26H_2_O, P: Portlandite (Ca(OH)_2_), C: Calcite (CaCO_3_), L: Larnite (Ca_12_Al_14_O_33_), and M: Mayenite (Ca_12_Al_14_O_33_).

The microscopic observation was performed by using the XRD curve to analyze hydration characteristics in the BOF slag mortar. Most substances showing peak intensity were almost identical to the 100% OPC mix of OFA0, such as ettringite, portlandite, calcite, larnite, and mayenite, as shown in Figure 7. However, it seemed that the peak intensity for each hydrate was strongly dependent on mix types. It was seen that OFA30 had a higher peak intensity for portlandite, calcite, larnite, and mayenite phases, while OFA0 and OFA15 showed a lower peak of these hydrates. As observed in the XRD curve, calcite’s and portlandite’s higher peak intensities in OFA30 at 29°(2θ) seemed overlaid due to a simultaneously indicative angle.

Nevertheless, OFA30 had a higher intensity of hydrates than other mixes. The reactivity of SCMs with higher basicity can induce higher reactivity since hydrates’ formation is possible under alkali conditions. The basic coefficient (CaO+MgO+Al_2_O_3_/SiO_2_) of PFA from the XRF test was 0.35, while those of OPC, FNS, and BOF slag were 4.16, 0.68, and 4.62, respectively. Based on Table 2 in Section 2.1, it is evident that PFA had the lowest alkalinity and the highest fineness among the four types of raw materials used in this study. Thus, the OFA30 mix had the lowest pH value compared to other mixes by partially substituting the PFA binder. It tends to have low carbonation resistance and hydration heat evolution, and clinker in OFA30 cannot suitably activate. Although FNS had acidic characteristics, the detected main components of OFA15 were almost unchanged compared to OFA30. The clinker of FNS was not identified, which indicates that some clinker of FNS was attributed to the hydration process. In the case of OFA0, however, the peak intensity of BOF slag clinker (i.e., larnite and mayenite) was depleted compared to OFA15 and OFA30. This is presumably attributed to the high hydration temperature and pH value, which can be accelerated by BOF slag hydration, as Wang et al. [27] demonstrated.

#### 3.3.2. Micro CT Analysis

**Figure 8:**
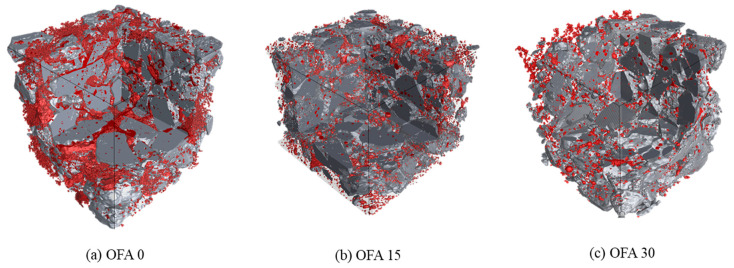
The 3D tomography of mortar samples: (**a**) OFA0, (**b**) OFA15, (**c**) OFA30.

A 3D tomography image of mortars is shown in Figure 8. As shown in Figure 8, the red-colored region demonstrates the pores formed at the vicinity of the BOF aggregate. It can be seen that the void amount of OFA0 is significantly higher than that of OFA15 and OFA30. The quantitative values of the porosity were 7.25, 1.70, and 2.57% for OFA0, OFA15, and OFA30. The high pore content in OFA0 mortar was caused by the expansion of the BOF aggregate. Monshi and Asgarani [28] defined that the three CaO contained in the BOF aggregate reacted in an alkaline environment. When free CaO hydrates, its volume and increases the interfacial zone between the cement paste and the aggregate. On the other hand, the test results showed that the incorporation of PFA or FNS relieved the volume expansion of BOF. It can be explained by the reduction of the average fineness of the binder due to the use of SCMs, which decrease of the amount of OH to react with free CaO. Therefore, using BOF aggregate together with PFA and FNS can be a way to eliminate the disadvantages of BOF, such as volume expansion.

## 4. Conclusions

In this study, the soundness reactivity of BOF slag mortars was investigated by setting time, compressive strength, length change measurement, XRD analysis, and X-ray CT. Detailed conclusions from these experiments are as follows.

(1) The incorporation of PFA can increase the fluidity of BOF mortar due to the ball-bearing effect. However, the VICAT test results showed a delayed setting of fresh mortar mixed with PFA and FNS.
(2) It was confirmed that BOF-incorporated mortar showed lower strength than natural sand mortar, especially at the early curing ages. The use of PFA or FNS can compensate for the strength reduction caused by the BOF aggregate.
(3) The length change of OFA0 was significantly higher than that of OFA15 and OFA30. With a higher use of OPC, a more undesirable hydraulic reaction would occur in the BOF slag mix, and, in turn, cause a soundness volume change.
(4) From XRD analysis, most substances showing their peak intensity were almost identical to ordinary mortar using natural sand. However, it seemed that the peak intensity for each hydrate was strongly dependent on mix types. It was seen that OFA30 had a higher peak intensity for portlandite, calcite, larnite, and mayenite phases while OFA0 and OFA15 showed a lower peak of these hydrates.
(5) The expansion aspect was visually measured by X-ray CT analysis. The bulk expansion was detected in OFA0, while there was no adverse effect in using FNS and PFA with the BOF aggregate. The decrease in the alkalinity of the pore solution by using SCMs may reduce the amount of OH to react with free CaO, consequently reducing the volume expansion of BOF aggregate.

## Figures and Tables

**Table 1:** Classification of steel slag.

Steel slag	**Type of Process**	**Type of Slag**		**Standard**
	Blast furnace slag	Air-cooled slag		[14]
		Water-cooled slag		
	Steel-making slag	Converter slag		-
		Electric arc furnace slag	Oxidation slag	[15]
			Reduction slag	

**Table 2:** Chemical and physical properties of raw materials (%).

	CaO	SiO_2_	Al_2_O_3_	MgO	Fe_2_O_3_	SO_3_	K_2_O	P_2_O_5_	TiO_2_	Loss on Ignition	Density (g/cm^3^)	Fineness (cm^2^/g)
OPC	66.98	17.43	3.97	1.60	4.16	3.41	1.23	0.14	0.27	0.4	3.14	3539
PFA	3.93	65.48	18.48	0.64	5.81	0.80	1.45	0.27	1.12	1.95	2.20	3850
FNS	6.28	48.23	3.59	23.01	15.76	0.50	0.09	0.20	0.11	0.02	3.12	3400
BOF slag	44.95	11.60	6.50	2.19	28.12	0.18	0.15	1.37	0.60	0.32	3.27	-

**Table 3:** Mix proportion of mortars (kg/m^3^).

	OPC	PFA	FNS	BOF Slag	Sand	Water
OFA0	571	-	-	700	700	257
OFA15	362	86	86	700	700	257
OFA30	362	171	-	700	700	257

**Table 4:** X-ray Source.

	Transmission Target	Directional Target	High Power Target
Voltage	30~120 kV	30~225 kV	20~320 kV
Forcal Spot size	0.4 μm	6 μm	400 μm

**Table 5:** Scan condition.

Diameter (mm)	Voltage (kVp)	Current (mA)	Transmission Time (s)	Source-Object Distance (mm)	Pixel Pitch (mm)
100	200	0.8	1	316	0.106488

**Table 6:** Test results of flow and setting time.

Specimen	Flow (mm)	Setting Time (min)	
		Initial Set	Final Set
OFA0	170	195	245
OFA15	176	260	382
OFA30	181	314	454

## Data Availability

The data presented in this study are available on request from the corresponding author.
